# Conventional manual technique of post placental IUD insertion versus intra-cesarean post placental introducer withdrawal IUD insertion technique: a new standardized technique for IUD insertion during cesarean section: a randomized controlled trial

**DOI:** 10.1186/s12884-023-05777-1

**Published:** 2023-06-26

**Authors:** Mostafa Seleem, Mona M. Sedik, Azza M. M. Megahed, Hala Nabil

**Affiliations:** Kasr Al-Ainy Maternity Hospital, Cairo University, Cairo, Egypt

**Keywords:** Contraceptive device, Cesarean section, Insertion technique, Complications

## Abstract

**Background:**

Inserting IUDs during cesarean section reduces the need for more manipulation and discomfort. The current conventional manual technique for IUD insertion during cesarean section is not standardized with many modifications and high rates of expulsion, displacement, missed threads, and discontinuation. This study aims to find a standard technique for IUD insertion during cesarean section with the least possible problems, especially displacement and missed threads.

**Methods:**

A randomized controlled study was conducted at Kasr Al-Ainy Maternity Hospital, Cairo University, Egypt. The study was performed over 12 months, from September 2020 to September 2021. Two groups of patients were selected; each group included 420 patients with a desire for IUD insertion during cesarean section. Group (A) (Control group) was subjected to a post-placental intrauterine device (Copper T380) during cesarean section using a conventional manual method; Group (B) (Study group): the IUD (Copper T380) was placed at the top of the uterine fundus using a new technique (intra-cesarean post placental introducer withdrawal IUD insertion technique).

**Results:**

There was a significant statistical difference between the two groups regarding displacement of the IUDs at the end of puerperium, at 6 months, non-visibility of IUD threads, and continuation of use with *p*-value < 0.05. There was no significant statistical difference in the term of duration of surgery.

**Conclusion:**

The new technique of post-placental IUD insertion can be the standard technique of intra-cesarean section IUD insertion as it is associated with more favorable outcomes among the included women in the form of lower incidence of IUD displacement, non-visibility of IUD threads, and higher rates of continuation without increasing the duration of surgery as compared with the conventional manual technique.

**Trial registration:**

ClinicalTrial.gov ID: NCT05788354, registration date: 28/03/2023 (retrospectively registered).

**Supplementary Information:**

The online version contains supplementary material available at 10.1186/s12884-023-05777-1.

## Introduction

In Egypt, an intrauterine contraceptive device (IUD) is commonly used more than any other kind of contraceptive [1]. Despite IUD side effects which may include excessive monthly flow, pain, displacement, infection, and expulsion, it is still the most often used contraceptive [2, 3].

The most upsetting risk is the displacement of the IUD, particularly if it occurs extra-uterine. Displacement of IUDs raises the possibility of unintended pregnancy and the complications of possible surgical interventions. To decrease the possibility of displacement, it is crucial to place IUDs at the right time and with the right procedure [4]. The best time to insert the IUD after a cesarean section is debatable. Gynecologists prefer to insert IUDs either immediately after puerperium (42 days), or after three months [5]. Inserting IUDs during cesarean section reduces the need for more manipulation and discomfort.

Moreover, women now have a strong desire to start using contraceptives. The current conventional manual technique for IUD insertion during cesarean section is not standardized with many modifications and variable rates of displacement and expulsion (5.23–22%), missed threads (0–72%), and discontinuation (0–15%) [5–7]. This study aims to find a standard technique for IUD insertion during cesarean section with the least possible problems, especially displacement, and missed threads.

## Material and method

### Study design

This is a prospective randomized controlled study that was conducted to compare between the conventional (manual) technique of post-placental IUD insertion and a new technique (intra-cesarean post-placental introducer withdrawal IUD insertion technique) for IUD insertion during cesarean section regarding side effects and complications.

### Study setting and duration

This study was performed at the Department of Obstetrics and Gynecology, Kasr Al-Ainy Hospital, Cairo University, Egypt. The study was conducted over 12 months, from September 2020 to September 2021.

### Study subjects

The study included 840 pregnant women to whom IUD was planned to be inserted during cesarean section upon their requests. The cases were randomly divided into two equal groups according to the technique of insertion as follows: Group A (Control group): Included 420 pregnant women where the IUD was placed at the top of the uterine fundus manually (Copper T380) using the conventional method of post placental IUD insertion; Group B (Study group): Included 420 pregnant women where the IUD was placed at the top of the uterine fundus (Copper T380) using the new technique of IUD insertion.

### Inclusion criteria

We included pregnant women whose age was between 18-45 years old and attending for elective or emergency cesarean section. The patients desired immediate IUD insertion.

### Exclusion criteria

We excluded women with the upper segment or classical cesarean scar, cesarean for placenta previa or placenta accreta, and evident infections during cesarean section as chorioamnionitis, uterine anomalies, uterine myomas, and bleeding tendency.

### Method of randomization

Participants were randomly allocated to the two groups using opaque envelopes. Then, the envelopes were opened sequentially immediately before IUD insertion to maintain concealment. After that, the randomization list was generated using a “computer software” by a statistician not otherwise included in this study. The participant’s allocation is based on the 1:1 ratio. Then, the investigators enrolled participants and assigned them to interventions. A record of the type of chosen intervention and the insertion method was kept to facilitate analyses based on the intention to treat by protocol. Finally, participants were blinded to the group allocation.

### Patients consent

Written informed consent was obtained from all participants before inclusion in the study, explaining the value of the study plus the procedures that were commenced.

### Ethical consideration

The study design was approved by the Research Scientific Ethical Committee (RSEC), Department of Obstetrics and Gynecology, Faculty of Medicine, Cairo University, Egypt (reference number I20015). Confidentiality and personal privacy were respected at all levels of the study. Patients felt free to withdraw from the study at any time without any consequences. The collected data were not and will not be used for other purposes. All methods were carried out per relevant institutional guidelines and regulations.

### Intervention

A lower-segment cesarean section was carried out for all participants after a full clinical assessment. Following placental delivery Copper T380 IUDs were placed in the uterine cavity as follows:

### Group A (control group): (*n* = 420)

First, we performed time out and verified that there were no contraindications to IUD placement; Second, after package opening, we removed the IUD from the introducer and trimmed IUD threads to 12 cm; Third, we grasped the IUD firmly along the stem of the device; Fourth, we stabilized the uterus using the non-dominant hand or with the aid of an assistant and advanced the IUD through the hysterotomy to the fundus; Fifth, we removed the hand and directed the IUD threads manually into the cervix; and Finally, we closed the uterine incision, took care not to incorporate the IUD threads.

### Group B (study group): (*n* = 420)

In our new technique, we applied the same idea of the withdrawal technique used for IUD insertion in gynecology. In this technique, the intoducer is used to push the IUD unfolded arms (after its release) to place it high up in the uterine fundus and withdraw it without pulling the IUD with it downwards. This is because the diameter of the introducer (3.8 mm) is wider than the diameter of the IUD stem (3 mm), so the introducer is not grasping on the IUD stem. In our new technique, the arms of the IUD remain unfolded through the steps of the technique.

**Fig. 1:**
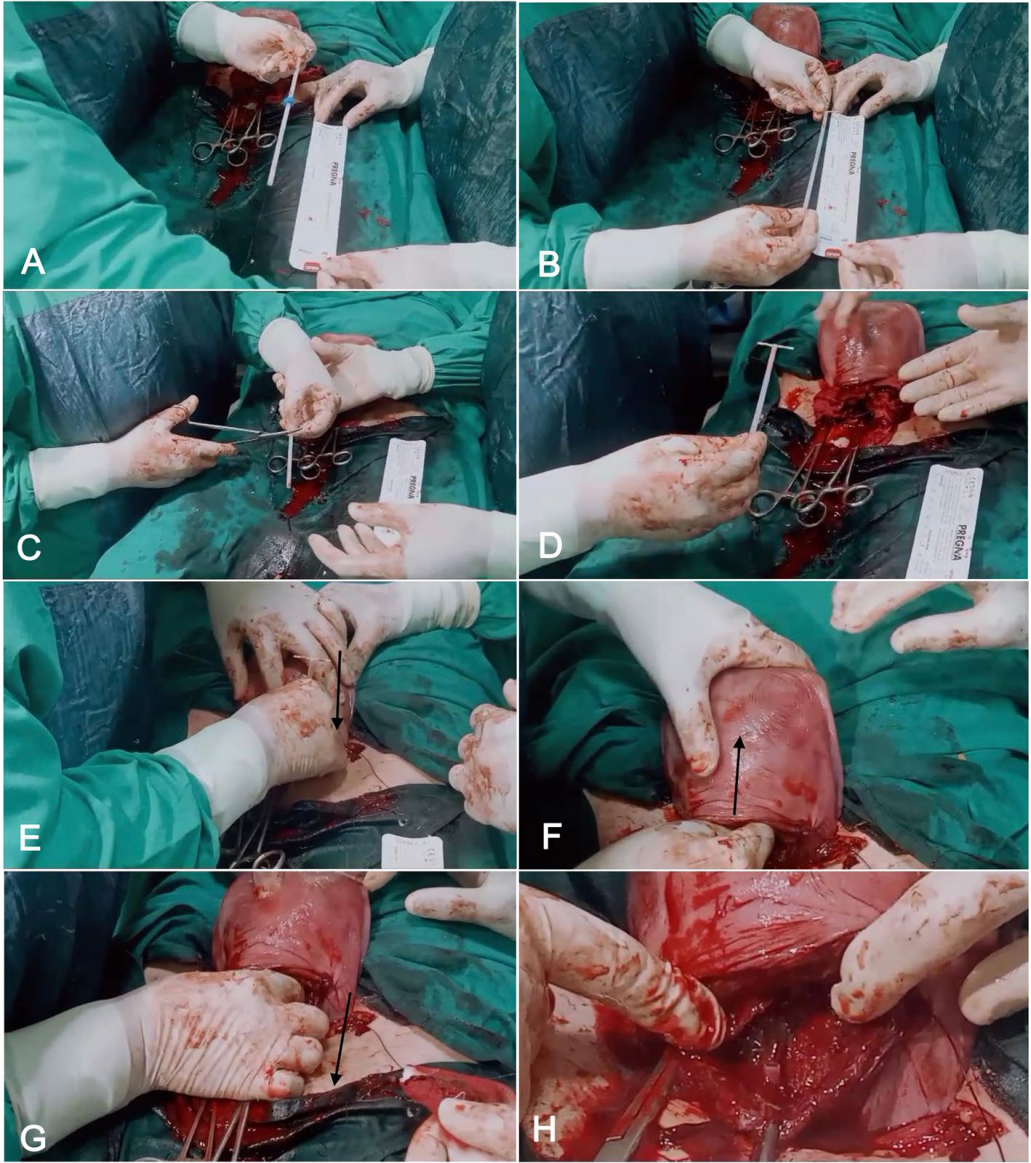
Steps of the new technique for intra-cesarean Copper T380 IUD insertion. **A** IUD inside the introducer with the blue flange in place. **B** Measuring the 12 cm length of the introducer after removal of the blue flange. **C** Trimming the introducer with the IUD threads to 12 cm. **D** The trimmed introducer with the IUD stem and threads inside and with the IUD arms unfolded. **E** The introducer is pushed gently in the cervical canal (black arrow). **F** The introducer is pushed upwards to the fundus (black arrow) with the non-dominant hand stabilizing it. **G** The introducer is pulled gently downwards to pass through the cervical canal down to the vagina (black arrow). **H** The upper part of the introducer seen above the internal cervical os with the IUD threads inside

First, we performed time out and verified that there were no contraindications to IUD placement; Second, after the package opening, we removed IUD from the package and trimmed the IUD introducer with the IUD threads inside to 12 cm after the removal of the distance marker (The collar or the blue flange) of the introducer (We trimmed the introducer to be only 12 cm so it can pass easily through the cervix without hitting the posterior vaginal wall in case the cervix is directed acutely posterior as in elective cesarean section cases. Moreover, we trimmed the IUD threads to ensure their passage through the external os of the cervix without the need to shorten them after the closure of the abdomen); Third, we held the uterus, stabilized it with the non-dominant hand, and inserted the introducer with the IUD threads inside first downwards through the cervical canal before pushing it up to put it firmly against the endometrium of the uterine fundus. Then, we pulled it gently down through the cervical canal to the vagina while pressing on the fundus with the non-dominant hand, thus ensuring that the IUD is kept in the fundus and that the IUD threads are in the cervical canal. The pulling of the introducer will not withdraw the IUD as it is now grasped by the non-dominant hand. Even if the non-dominant hand is grasping both the IUD and the introducer, the introducer will not pull on the IUD downwards with it as it does not grasp on it. Finally, we closed the uterine incision, and took care not to incorporate the introducer or the threads. Next, we removed the introducer gently manually from the vagina after the closure of the skin and ceiling of the wound (Fig. 1). An additional movie file shows the technique in more detail (see Additional file 1).

### Measured outcomes

#### Primary outcome

Intrauterine displacement of the IUD (by vaginal ultrasound) by an independent assessor at the end of puerperium and six months following up the distance from the top of the uterine cavity to the IUD, which should be < 3 mm).

### Secondary outcome parameters

◦ Follow up for:
  Missed or non-visibility of IUD threads by speculum examination (at the end of puerperium and six months).
  Discontinuation of the method (at six months) by questionnaire assessment.
  Duration of surgery from the skin incision till the closure of skin wound (time calculation in minutes).

### Statistical analysis of data

The collected data were handled and studied using SPSS (Statistical Package for Social Sciences) version 27. Mean (X) and standard deviation (SD) were used for normally distributed data, while for skewed data, median (Med) and interquartile range (IQR) were used. For qualitative data, the frequency with percentage (%) was used. Analytical or inferential statistics included Fisher’s Exact Test: It was used to compare two or more groups regarding one qualitative variable. It was used instead of Chi-Square (χ2) test when the assumption that at least 80% of the expected frequencies are greater than five was violated. Independent samples t-test was used for continuous data to test for significant differences between two normally distributed groups. Assumptions of normality in each group and homogeneity of variances were verified using the Shapiro-Wilk test and Levine’s test, respectively. The Mann–Whitney U test was used for continuous data to test for significant differences between two abnormally distributed groups. Significant test results are quoted as two-tailed probabilities. For all the tests mentioned above, the level of significance was tested, expressed as the probability of (*p*-value), and the results were explained as follows: non-significant if the *p*-value is > 0.05, significant if the *p*-value is ≤ 0.05, and highly significant if the *p*-value < 0.001.

### Sample size

As considered the primary outcome, sample size calculation was conducted to compare the IUD displacement rate between IUD insertion using the conventional technique and IUD insertion using the new technique. The calculation was performed based on comparing two proportions from independent samples using the Fisher Exact test, the α-error level was fixed at 0.05, and the power was set at 95%. Accordingly, the optimum minimal sample size should be 420 participants in each group.

## Results

Participants were recruited at Kasr Al-Ainy Hospital, Cairo University, Egypt, over 12 months from September 2020 to September 2021.

**Fig. 2:**
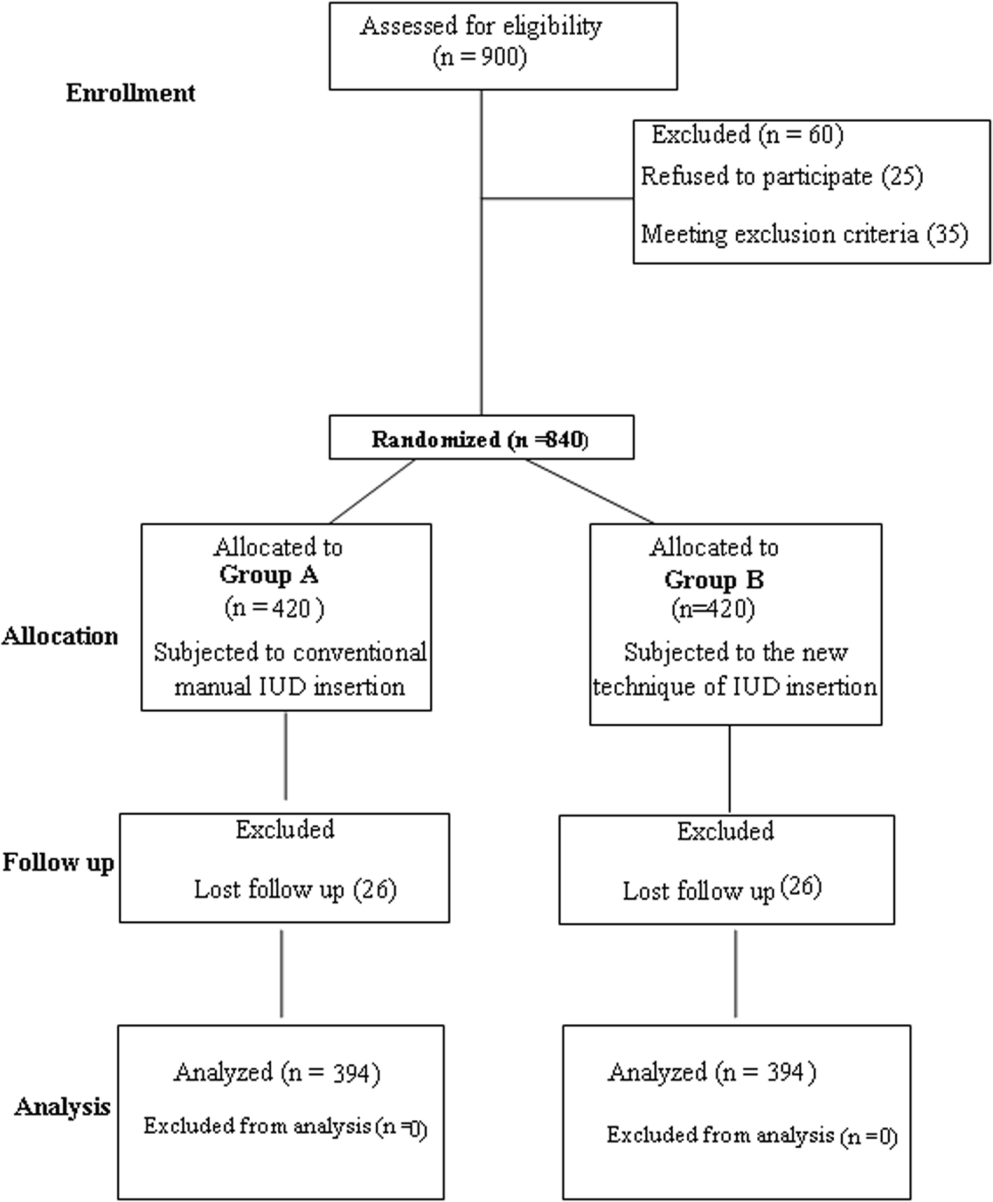
CONSORT flow chart of the study design

After the exclusion of cases that refused to participate or who met exclusion criteria, the rest of the eligible cases were randomly divided into two equal groups (each of 420 women): The conventional post-placental IUD insertion technique (Group A), and the new technique (Group B). The excluded cases due to lost follow-up were equal in the two groups (26), and finally, 394 cases in each group were analyzed (Fig. 2). All eligible cases were followed up for six months. The study ended with the last follow-up visit of eligible cases (Tables 1 and 2).

**Table 1:** Baseline characteristics data of included study groups

Baseline data	Group A Conventional technique (*N* = 394)	Group B New technique (*N* = 394)	*P*-value
**Age (years)** (Median and Range)	25 (20–32)	25 (22–33)	0.085
**BMI (kg/m²)** (Median and Range)	30 (26–35)	30 (25–34)	0.127
**Parity** (Median and Range)	2 (1–3)	2 (1–4)	0.410
**First time CS**	106 (26.9%)	115 (29.2%)	0.763
**Previous 1 CS**	114 (28.9%)	103 (26.1%)	
**Previous 2 CS**	89 (22.6%)	86 (21.8%)	
**Previous 3 CS**	56 (14.2%)	54 (13.7%)	
**Previous 4 CS**	29 (7.4%)	36 (9.1%)	

There was no significant statistical difference regarding age, BMI, parity, and No of CS *p*-value > 0.05 (Table 1)

**Table 2:** Outcome data of included study groups

Outcome data	Group A Conventional technique (*N* = 394)	Group B New technique (*N* = 394)	*P*-value
**Displacement of IUD at the end of puerperium**	61 (15.5%)	39 (9.9%)	0.019
**Displacement of IUD at 6 months**	79 (20.1%)	47 (11.9%)	0.002
**Non-visibility of IUD threads at 6 months (missed threads)**	38 (9.6%)	14 (3.6%)	0.001
**Continuation**	315 (79.9%)	347 (88.1%)	0.002
**Duration of surgery (min)** (mean and SD)	57.2 ± 13.3	58.4 ± 15.9	0.830

There was a significant statistical difference regarding the displacement of the IUDs at the end of puerperium, at 6 months, non-visibility of IUD threads, and continuation with *p*-value < 0.05. There was no significant statistical difference concerning the duration of surgery *p*-value = 830 (Table 2)

## Discussion

The ideal time to place an IUD following a cesarean birth is still up for discussion. Recent studies have reported inserting the IUD as soon as possible after birth (within 10 min of placental delivery) to maximize the usage of trustworthy, efficient, and long-lasting contraceptive techniques, especially in countries with high populations and at a time when women are highly motivated for contraception [8–11].

Till now, there is no standardized technique for IUD insertion during cesarean section. The conventional technique of IUD insertion during cesarean section, as described in previous studies, is based on the manual placement of the IUD in the uterine fundus and manual guide of the IUD threads through the cervix [8–11]. In some studies, they used long curved Kelly’s placental forceps, ovum forceps, or the IUD introducer to ensure the placement of the IUD in the uterine fundus [12, 13]. In another study, they used the introducer to guide the threads in the cervix without a clear description of this step [13]. None of the previous studies studied the value of technique modifications on IUD side effects and complications. Therefore, it is clear that there is a need for a standard technique for IUD insertion during cesarean section with the least possible side effects and complications.

Our study compared the conventional manual technique to a new technique (intra-cesarean post-placental introducer withdrawal IUD insertion technique). This is the first study that describes and outlines this technique in detail and its advantage compared to the conventional post-placental manual IUD insertion technique.

The rationale of our technique is to use the introducer to ensure placing the IUD in the uterine fundus and ensuring that its threads pass through the cervix to the vagina. The conventional manual insertion technique of grasping the IUD from the stem does not ensure that the arms of the IUD are firmly in contact with the uterine fundus, while pushing the IUD unfolded arms with introducer up to the fundus ensures that. Additionally, we must note that pushing the IUD differs from grasping and releasing it. Putting a grasped IUD in the uterine fundus may be followed by some degree of pulling the IUD downward during the withdrawal of the grasping hand (or even a grasping instrument) due to the narrowness of the available cavity that creates some negative pressure and due to some inability to completely ungrasp the IUD. In contrast, withdrawal of the introducer will not be followed by pulling forces to the IUD downwards as the introducer now has no attachment to the IUD, as usually happens in routine IUD insertion withdrawal techniques used in gynecology.

In another study, they used the introducer during the insertion technique, but they did not trim it or trim the threads and they put the IUD first in the fundus before passing it through the cervix. We can expect that they have faced difficulty in passing the introducer through the cervix as in this way as the length of the uterine cavity may not allow easily for the entry of the whole introducer before pushing it through the cervix, and that they faced resistance by the posterior vaginal wall in case the cervix is directed posterior. Furthermore, in this study, they compared intra-cesarean insertion with interval insertion [13].

IUD displacement is the major problem of IUD usage, leading to other problems such as unwanted pregnancy, expulsion, bleeding, and uterine colics [14–16]. In our study, the incidence of IUD displacement in the new technique was 9.9% at the end of puerperium, and the cumulative percentage at 6 months was 11.9%. While in the conventional post-placental IUD insertion technique, the percentage of IUD displacement at the end of puerperium was 15.5%, and the cumulative percentage of IUD displacement at 6 months was 20.1% which was statistically significantly lower in the new technique. In another study, the percentage of intrauterine displacement using the conventional method was 10% after 1 year; however, the number of cases in this study was less than 100, while in our study, the number was 394. In addition, this study compared the conventional manual technique to interval IUD insertion [13].

Fixation of the IUD after placement of it in the uterine fundus will lead to less liability for displacement [17]. GyneFix*®* CS for intra-cesarean insertion by Wildemeersch and Gyne-T 380 postpartum IUD has low displacement and expulsion rates (2.7% and 9.5%, respectively) [18].

However, we aimed to standardize a technique that can be used in developing countries with limited resources using the commonly available types of IUDs and the regular tools for IUD insertion provided with each package. Moreover, suturing and fixing the IUD to the uterus may increase the risk of bleeding, prolongation of operative time, and undue surgical difficulty. Still, there will be a need for studying the additive value of fixing the IUD to this new technique as previous studies applied fixation of the IUD with conventional manual technique.

Thread visibility may be a problem with post-placental IUD placement. Non-visibility of IUD threads makes the dilemma of missed IUD difficult to extract. In our research, the new method group had a proportion of missing IUDs threads (after 6 months) of 3.6%, which was statistically significantly lower than the conventional technique (9.6%). The FIGO project reported the rates of absent threads in around one-third of women (visible in two-thirds), although this was for both vaginal and cesarean births [19]. In one study, the incidence of missed IUD threads after one year following post placental conventional technique was 48.15% [9]; in another, the incidence was 13% after 12 months [13]. In another study, although they used ring forceps to guide the IUD threads through the cervical canal, the incidence of non-visibility of the threads was 44%. In a systematic review that was updated in 2017, the incidence of missed IUD threads ranged from zero to 72% but with different types of IUDs and some studies had a small number of cases [5]. In a study by Singal et al., they used copper T 380 A IUDs in 300 cases, and the percentage of missed threads after 6 months was 16% [6]. We can conclude that one of the major advantages of our technique is the use of the introducer, as we described, to ensure the passage of the IUD threads through the cervical canal to be visualized in the vagina.

In our study, we took the continuation of the IUD usage after 6 months as a marker for acceptance, side effects, and complications. The continuation of IUD usage was higher in the new technique group than in the conventional technique group (88.1% versus 79.9%, with a highly significant statistical difference). In another study in which they used the introducer to put the IUDs in the fundus and ring forceps to guide the threads in the cervical canal, the continuity after 6 months was 83%, considering that they used different types of IUDs [7].

Finally, we studied the duration of surgery between the two groups to know if our new technique added more significant time for the surgical procedure, and there was no significant statistical difference between the two groups as regards the duration of surgery. However, more trials of the technique with different types of IUDs and different levels of obstetricians’ training are needed.

## Conclusion

Our new technique can be the standard technique for intra-cesarean section IUD insertion as it is associated with a lower incidence of IUD displacement, non-visibility of IUD theads, and a higher rate of continuation without increasing the duration of surgery as compared with the conventional technique. In addition, the standardization of the technique is essential for future studies so that the results can be compared and reproduced. Any future new modifications of the technique should be applied to a standard technique for the reliability of the value of these modifications.

## Supplementary Information

**Additional file 1.**

## Data Availability

The datasets used and/or analyzed during the current study are available from the corresponding author upon reasonable request.
